# KCNMA1 Encoded Cardiac BK Channels Afford Protection against Ischemia-Reperfusion Injury

**DOI:** 10.1371/journal.pone.0103402

**Published:** 2014-07-29

**Authors:** Ewa Soltysinska, Bo Hjorth Bentzen, Maria Barthmes, Helle Hattel, A. Brianne Thrush, Mary-Ellen Harper, Klaus Qvortrup, Filip J. Larsen, Tomas A. Schiffer, Jose Losa-Reyna, Julia Straubinger, Angelina Kniess, Morten Bækgaard Thomsen, Andrea Brüggemann, Stefanie Fenske, Martin Biel, Peter Ruth, Christian Wahl-Schott, Robert Christopher Boushel, Søren-Peter Olesen, Robert Lukowski

**Affiliations:** 1 The Danish National Research Foundation Centre for Cardiac Arrhythmia, University of Copenhagen, Copenhagen, Denmark; 2 Department of Biomedical Sciences, Faculty of Health and Medical Sciences, University of Copenhagen, Copenhagen, Denmark; 3 Center for Integrated Protein Science Munich (CIPSM), Ludwig-Maximilians-Universität, Munich, Germany; Department of Pharmacy, Center for Drug Research, Ludwig-Maximilians-Universität, Munich, Germany; 4 Nanion Technologies GmbH, Munich, Germany; 5 Department of Biochemistry, Microbiology and Immunology, Faculty of Medicine, University of Ottawa, Ottawa, Canada; 6 Department of Biomedical Sciences, Core Facility for Integrated Microscopy, Faculty of Health and Medical Sciences, University of Copenhagen, Copenhagen, Denmark; 7 Department of Physiology and Pharmacology, Karolinska Institutet, Stockholm, Sweden; 8 Department of Pharmacology, Toxicology and Clinical Pharmacy, Institute of Pharmacy, University of Tübingen, Tübingen, Germany; Northwestern University, United States of America

## Abstract

Mitochondrial potassium channels have been implicated in myocardial protection mediated through pre-/postconditioning. Compounds that open the Ca^2+^- and voltage-activated potassium channel of big-conductance (BK) have a pre-conditioning-like effect on survival of cardiomyocytes after ischemia/reperfusion injury. Recently, mitochondrial BK channels (mitoBKs) in cardiomyocytes were implicated as infarct-limiting factors that derive directly from the KCNMA1 gene encoding for canonical BKs usually present at the plasma membrane of cells. However, some studies challenged these cardio-protective roles of mitoBKs. Herein, we present electrophysiological evidence for paxilline- and NS11021-sensitive BK-mediated currents of 190 pS conductance in mitoplasts from wild-type but not BK^−/−^ cardiomyocytes. Transmission electron microscopy of BK^−/−^ ventricular muscles fibres showed normal ultra-structures and matrix dimension, but oxidative phosphorylation capacities at normoxia and upon re-oxygenation after anoxia were significantly attenuated in BK^−/−^ permeabilized cardiomyocytes. In the absence of BK, post-anoxic reactive oxygen species (ROS) production from cardiomyocyte mitochondria was elevated indicating that mitoBK fine-tune the oxidative state at hypoxia and re-oxygenation. Because ROS and the capacity of the myocardium for oxidative metabolism are important determinants of cellular survival, we tested BK^−/−^ hearts for their response in an *ex-vivo* model of ischemia/reperfusion (I/R) injury. Infarct areas, coronary flow and heart rates were not different between wild-type and BK^−/−^ hearts upon I/R injury in the absence of ischemic pre-conditioning (IP), but differed upon IP. While the area of infarction comprised 28±3% of the area at risk in wild-type, it was increased to 58±5% in BK^−/−^ hearts suggesting that BK mediates the beneficial effects of IP. These findings suggest that cardiac BK channels are important for proper oxidative energy supply of cardiomyocytes at normoxia and upon re-oxygenation after prolonged anoxia and that IP might indeed favor survival of the myocardium upon I/R injury in a BK-dependent mode stemming from both mitochondrial post-anoxic ROS modulation and non-mitochondrial localizations.

## Introduction

Ischemia and post-ischemic reperfusion induce cardiac damage [1]. Brief episodes of ischemia prior to or after the prolonged ischemic insult have been shown to be very effective in rendering the heart less susceptible to damage [2], [3]. The infarct-reducing effects via ischemic pre-conditioning (IP) are mediated by multiple factors, which, at least in part, signal through potassium channels present at the inner mitochondrial membrane (IMM) of the cardiomyocyte (CM) [4]. Opening of mitochondrial K_ATP_ (mito_KATP_) channels has been shown to modulate mitochondrial bioenergetics and to inhibit formation of the mitochondrial permeability transition pore (mPTP), which is a major determinant of irreversible injury during reperfusion when oxygen is reintroduced and the IMM potential is regenerated [5]–[8]. However, their cardio-protective roles in the mitochondrion have also been questioned [9]. In addition to mitoK_ATP_, intracellular Ca^2+^-activated potassium channels of big-conductance (BK) [10] first recognized in a human glioma cell line [11] have been identified in mouse CMs [12] at the IMM (mitoBK) [13]–[15], but not in the sarcolemma [15], [16]. Experiments using isolated ventricular CMs from rat and guinea pig hearts suggest that opening of mitoBK prevents mitochondrial matrix Ca^2+^ overload and attenuates reactive oxygen species (ROS) production, pathological hallmarks for mPTP formation and cell death, upon reperfusion of the heart [16]–[18]. MitoBK activation may thus confer cardio-protection in a manner similar to but independent of mitoK_ATP_ [19], but to this end its function at physiological conditions is unclear. Physiological and pharmacological properties of mitoBK channels purified from cardiac mitochondria indicate that their properties are comparable to plasma membrane BK channels [15], [20]–[23]. Importantly, BK channel openers (NS1619 and NS11021) applied at the onset of reperfusion are cardio-protective independent of changes in K_ATP_ activity or hemodynamic alterations [24]–[26]. Cardio-protection was sensitive to the BK blocker paxilline and could be observed for 24 hours upon pretreatment [24]. Additionally, accessory β1-subunits of BK that modulate kinetics and Ca^2+^-sensitivity of the channel were identified in mitochondria of CMs and their knock-down abolished infarct-limiting effects of the phosphodiesterase-5 inhibitor sildenafil [13]. A combination of yeast two-hybrid and immune-cytochemical assays provided further evidence for an IMM complex enclosing BK β1-subunits and cytochrome c oxidase I indicating direct recruitment of multimeric BK channel proteins to the oxidative phosphorylation machinery [23]. A number of additional studies have reported the involvement of BK in the signaling pathway of agents leading to cardio-protection. This list includes among others adenosine [27], κ-opoid receptor agonist [28], β-estradiol [23], and desflurane [29], [30]. These agents protected the heart from I/R injuries when applied prior to ischemia, and the protection was attenuated by co-administration of toxins, such as paxilline, inhibiting BK, thereby strongly supporting the notion that pro-survival pathways promote BK activity. However, these conclusions have been recently challenged since the widely used BK opener NS1619 might have additional effects unrelated to mitoBK channels [31]–[33]. Furthermore, IP or anesthetic preconditioning (APC) protocols efficiently protected BK-negative hearts from I/R injury [34], although, beneficial effects of APC were sensitive to paxilline in the absence of BK. Finally, it has been suggested that protection via BK and NS1619 at I/R is a function of intrinsic cardiac neurons [35], but it remained largely elusive how the protective signals would be transmitted to the CM.

In contrast, in the presented study, we show immune blots supported by electrophysiological recordings that provide evidence for a paxilline- and NS11021-sensitive mitoBK channel at the IMM of CM. Ablation of KCNMA1 encoding the BK channel protein [36] resulted in elevated ROS release of CM mitochondria upon anoxia and reoxygenation and severe bioenergetic defects reflected by a reduced oxidative phosphorylation capacity (OXPHOS). Importantly, OXPHOS capacities of BK-deficient ventricular muscle fibres were attenuated at physiological/normoxic conditions and upon re-oxygenation after prolonged anoxia, whereas no gross abnormalities in mitochondrial ultra-structures i.e. matrix volume were detectable. Since a reduced bioenergetic reserve of CM mitochondria and high ROS at O_2_-reintroduction should render the heart more susceptible to damages, and IP might preserve ATP-synthesizing enzyme activities in the electron transport chain at I/R we studied whether BK^−/−^ hearts subjected to I/R injury would be protected by established IP paradigms. Together, our data suggest that IP requires mitoBK channels (and possibly non-mitochondrial channels present in e.g. the vasculature) to 1) enable a proper oxidative phosphorylation activity of mitochondria allowing ADP-stimulated respiration upon oxygen reintroduction to the myocardium, and 2) limit the I/R injuries to the myocardium possibly via dampening post-anoxic ROS generation.

## Research Design and Methods

### Experimental animals and ethic statements

All animals were bred in the animal facility of the Institute of Pharmacy, University of Tübingen and had free access to tap water and standard chow. Maintenance and procedures were conducted according to the local government’s committee on animal care and welfare in Tübingen, Copenhagen and Munich. The study conformed with the European Community Guidelines for the Care and Use of Experimental Animals, and was approved by the Animal Ethics Screening Committee of the Panum Institute (clearance number: 2011/0015-6221). BK channel knockout mice (BK^−/−^) that carry a global inactivation of the KCNMA1 gene were described earlier [36], [37]. For experiments organs, cells or organelles derived from BK^−/−^ animals were compared to wild-type mice (BK^+/+^) from the same litters on a mixed SV129/C57BL/6 (F1) genetic background since an efficient ischemic pre-conditioning of the SV129/C57BL/6 strain has been reported earlier [38].

### Purification of mitochondria from cardiac myocytes and immuno-blotting

The isolation of adult cardiomyocytes from BK^+/+^ and BK^−/−^ mouse hearts was performed as described previously [39] by a modified protocol of the Alliance for Cellular Signaling (AfCS) (AfCS procedure protocol PP00000125). Upon perfusion the hearts were minced into small pieces and myocytes were liberated by gently applying mechanical turbulence. A small fraction of cells was plated in 24-well format on laminin coated glass cover-slips to check for the quality and purity of the purification. The content of healthy rod-shaped CMs was usually >95%. Purifications with a lower mycoyte content or quality were not further processed. Mitochondria enriched fractions were generated according to Frezza et al. 2007 in less than 100 min from adult primary CMs [40]. In brief, the cells were washed and homogenized using a glass-teflon pestle based tissue-grinding system in IBc buffer (10 mmol/lTris–MOPS, 1 mmol/l EGTA/Tris, 0.2 M sucrose, pH 7.4) at 4°C. Cell debris and mitochondria were separated by three subsequent centrifugation steps at 700 g, 3000 g and 7000 g at 4°C for 10 min each, and then resuspended in a final volume of 30 µl in SDS lysis buffer. Western Blot analyses were performed only on freshly isolated mitochondrial lysates within 48 hours after the purification. For all experiments myocytes/mitochondria purified from 2–3 hearts per genotype were pooled.

Western Blot was performed as described previously [41]. The primary antibodies used were specific for BK (Alomone, #APC-107 (dilution 1∶250) and #APC-021 (dilution 1∶200)), COX4 (Cell Signaling, #4844 (dilution 1∶1000)) and hsp60 (Santa Cruz, #H-300 (dilution 1∶200)).

### Electrophysiological recordings from CM mitoplasts

Vital CMs were prepared by enzymatic digestion of mouse heart via retrograde perfusion through the aorta as previously described [42]. CM mitochondria were isolated [40] for preparation of mitoplasts by osmotic swelling in hypotone solution for 7 min. Subsequently isotonicity was restored [11]. Mitoplasts were freshly prepared directly before experiments, stored on ice and used within 2 h. Electrophysiological recordings were carried out using an internal solution containing (in mmol/L) 60 KF, 60 K-gluconate, 10 KCl, 10 HEPES, 200 sorbitol and an external solution containing (in mmol/L) 10 K-gluconate, 10 HEPES, 1 Ca-gluconate, 260 sorbitol. 10 µmol/l ruthenium red, 50 µmol/l propranolol, 10 µmol/l bepridil and 10 µmol/l cyclosporin A were freshly prepared and added to the internal and external solutions in order to block mitochondrial Ca^2+^ uniporter, inner membrane anion channels (IMAC), Slo2.2 and mPTP, respectively. Thereby, the identity of our cardiomyocyte mitoplast preparations could be confirmed since mitochondria-specific IMAC activity (data not shown) was reportedly blocked by propranolol [43]. Patch clamp experiments were performed using a Port-a-Patch system (Nanion) and a HEKA EPC-10 amplifier. NPC-1 chips with a resistance of 10–15 MΩ (diameter of the holes <1 µm) and an external salt bridge (3 mol/l KCl, 3% agarose) as ground reference were used. All recordings where done in whole mitoplast mode (capacitance of about 1 pF). Voltage pulses of 2 sec to 80 mV from a holding potential of −50 mV were applied every 5 sec. Each experiment consisted of three phases of 3 min duration. After the first control phase, NS11021 (10 µmol/l) was added to the external solution to ensure a significant increase in the open probability of BK [44], and 3 min later exchanged by an external solution containing paxilline (100 nmol/l).

Different amplitudes of single channel conductance were classified in each phase and experiment, and their frequency was calculated and normalized to the number of experiments. Multiple occurrences during one phase were counted as one.

### High-resolution respirometry on permeabilized myocardial muscle and soleus skeletal muscles

Surgically extracted hearts from BK^+/+^ and BK^−/−^ mice were prepared based on a previously published protocol [45]. In brief, hearts were placed in ice-cold preservation solution (BIOPS) containing 10 mmol/l CaK_2_EGTA buffer, 7.23 mmol/l K_2_EGTA buffer, 0.1 µmol/l free calcium, 20 mmol/l imidazole, 20 mmol/l taurine, 50 mmol/l 2-(N-Morpholino) ethanesulfonic acid hydrate (K-MES), 0.5 mmol/l dithiothreitol (DTT), 6.56 mmol/l MgCl_2_•6H_2_O, 5.77 mmol/l ATP, and 15 mmol/l phosphocreatine (pH 7.1). The left ventricle was sectioned and dissected free from surrounding connective tissue with needle forceps and 2 equal portions of ∼1–2 mg were isolated for mitochondrial respirometry. Prior to permeabilization the myocardial muscle bundles were gently dissected with two pairs of sharp forceps to achieve a high degree of fibre separation verified microscopically and by pallor. Chemical permeabilization of the sarcolemma was undertaken by incubation of the muscle fibre bundles with 20 µl of saponin (5 mg/ml) in 2 mL of BIOPS and stirred on ice for 20 min. Permeabilized samples were then rinsed with a mitochondrial respiration medium containing 0.5 mmol/l EGTA, 3 mmol/l MgCl_2_•6H_2_O, 60 mmol/l K-lactobionate, 20 mmol/l taurine, 10 mmol/l KH_2_P0_4_, 20 mmol/l HEPES, 110 mmol/l sucrose, and 1 g/l bovine serum albumin (pH 7.1). Immediately after rinsing, the left ventricular muscle bundles were blotted and measured for wet weight in a balance controlled for constant relative humidity, and each sample was then placed into a single chamber of the respirometer (Oxygraph, Oroboros Instruments, Innsbruck, Austria) containing mitochondrial medium (MiR05). Instrumental calibrations were performed to correct for back-diffusion of oxygen into the chamber, leak from the exterior, and sensor oxygen consumption. Oxygen flux was resolved by software allowing nonlinear changes in the negative time derivative of the oxygen concentration signal. Respirometry was performed at a chamber temperature of 37°C. Malate (2 mmol/l) was added to the chamber to prime the tricarboxylacid cycle and to achieve resting respiration in the absence of adenylates. Subsequent substrate titrations tested ADP-stimulated mitochondrial respiration catalyzing sequential redox reactions primarily coupled to the production of ATP via ATP synthetase (complex V). OXPHOS capacity of NADH dehydrogenase (Complex I (PCI)) was achieved with the addition of glutamate (10 mmol/l) followed by ADP (5 mmol/l). Maximal respiration and OXPHOS capacity (PCI+II) was stimulated with the addition of succinate (10 mmol/l) through complex II i.e. succinate dehydrogenase. To examine the effect of hypoxia/anoxia on the intrinsic function and viability of mitochondria during recovery from anoxia, the [O_2_] in the respirometer was reduced to zero by addition of N_2_ to the chambers. A first anoxia exposure of 5 min approximating the *in-vivo* ‘pre-conditioning’ condition was followed by re-oxygenation, and a second anoxia exposure of 60 min was applied and again followed by re-oxygenation. Subsequently, the integrity of the inner mitochondrial membrane was tested by titration of cytochrome c (10 µmol/l). Antimycin A (2.5 µmol/l) was then added to terminate respiration by inhibiting cytochrome bc1 complex (complex III), allowing for the determination of residual oxygen consumption in the oxygraph chamber. The maximal oxidative rate of cytochrome c oxidase (complex IV, or COX), the terminal respiratory chain complex that catalyzes the reduction of oxygen to water at cytochrome aa3 was assessed by addition of the redox substrates ascorbate (2 mmol/l) and N,N,N’,N’-tetramethyl-1,4-benzenediamine dihydrochloride (TMPD 5 mmol/l). Finally, to quantify and account for respiration due to auto-oxidation of TMPD, sodium azide (100 mmol/l) was added to the chamber.

For O_2_-respirometry of skeletal muscles a small portion of the biopsied soleus (∼2–3 mg) was placed into BIOPS medium (as above) followed by fibre separation and permeabilization with saponin. The muscle bundles were then rinsed, blotted and measured for wet weight in a balance. Muscle bundles were then transferred immediately into the respirometer containing the respiration medium (as above) at a chamber temperature of 37°C. All measurements were performed as described in detail for cardiac fibres, however instead of 60 min anoxia was maintained for 90 min with 100% nitrogen.

### Electron microscopy

The mice were pre-anaesthetised with inhalation of Halothane 3% (Halocarbon Laboratories, River Edge, NJ, USA). Anaesthesia was induced by doses of a 9∶1 mixture of Ketamine 25 mg/ml (Pfizer, Ballerup, Denmark) 90 mg/kg and Xylazine 20 mg/ml (Bayer, Leverkusen, Germany) 10 mg/kg. Vascular perfusion through the left ventricle of the heart was performed with 2% v/v glutaraldehyde in 0.05 M sodium phosphate buffer (pH 7.2) for 5 min. Briefly, following thoracotomy, a small incision was made of the apex of the heart, followed by insertion of a narrow cannula, which was clamped close to the apex. Following fixation the hearts were stored in the same fixative. Following isolation of suitable left ventricle specimen blocks proximal to the site of cannula insertion, the samples were rinsed three times in 0.15 M sodium cacodylate buffer (pH 7.2) and subsequently postfixed in 1% w/v OsO_4_ in 0.12 M sodium cacodylate buffer (pH 7.2) for 2 h. The specimens were dehydrated in graded series of ethanol, transferred to propylene oxide and embedded in Epon according to standard procedures. Sections, approximately 80 nm thick, were cut with a Reichert-Jung Ultracut E microtome and collected on copper grids with Formvar supporting membranes, stained with uranyl acetate and lead citrate, and subsequently examined with a Philips CM 100 TEM (Philips, Eindhoven, The Netherlands), operated at an accelerating voltage of 80 kV. Digital images were recorded an OSIS Veleta digital slow scan 2 k×2 k CCD camera and the ITEM software package.

### Measurement of reactive oxygen species production from isolated cardiac mitochondria

Hearts were removed as described above and placed in ice-cold isolation medium containing: sucrose (100 mmol/l), KCl (100 mmol/l), Tris-HCl (50 mmol/l), KH_2_PO_4_ (1 mmol/l), EGTA (100 µmol/l), BSA (0.1%) adjusted to pH 7.4. Mitochondrial isolation procedures were initiated within 5 hours of excision. Hearts were dissected free of connective tissue and fat, weighed and cut with small scissors into fine pieces followed by a three-step differential centrifugation procedure. In the initial isolation step bacterial protease (0.2 mg•ml^−1^) was added to the media. The final pellet was resuspended in preservation solution containing EGTA (0.5 mmol/l), MgCl_2_ (6.3 mmol/l), K-lactobionate (60 mmol/l), taurine (20 mmol/l), KH_2_PO_4_ (10 mmol/l), HEPES (20 mmol/l), sucrose (110 mmol/l), BSA (1 g/l), histidine (20 mmol/l), glutathione (3 mmol/l), leupeptine (1 µmol/l), glutamate (2 mmol/l), malate (2 mmol/l), Mg-ATP (2 mmol/l).

Mitochondrial ROS production and respiration were measured simultaneously using an Oroboros O2k oxygraph equipped with a flourometric sensor [46]. ROS production was detected using horseradish peroxidase (10 U) and Amplex Ultrared (20 µmol/l final concentration, Molecular Probes Invitrogen) added to each chamber. With a 3 µl mitochondrial suspension in the respirometer, the substrates pyruvate (5 mmol/l), malate (2 mmol/l), and succinate (10 mmol/l) were added to elicit ‘leak respiration’ and ROS production was recorded after stabilization of the signal. Subsequently, ADP (2 mmol/l) was added and oxygen in the chamber was set to ∼3 kPa using nitrogen gas. The chamber was closed and the ROS signal at ∼1.5–2 kPa oxygen was termed “pre-anoxia”. Mitochondria were then allowed to consume all oxygen in the chamber and then remained in anoxia for 20 minutes, whereupon oxygen was allowed back into the chamber and ROS-production was again recorded. The ROS signal was calibrated, adjusted for non-biological drift and quantified in connection with every experiment using a multiple-point standard curve with HCl stabilized stocks of H_2_O_2_. Oxygen consumption was recorded simultaneously using the same principles as with the permeabilized fibers.

### Isolated mouse heart Langendorff preparations

Animals were anesthetized subcutaneously by a mixture of midazolam (12.5 mg/kg), fluanisone (25 mg/kg), and fentanyl citrate (0.8 mg/kg), and anticoagulated by intraperitoneal injection of heparin (1,000 IE/kg). After induction of surgical anesthesia, the heart was excised through a median sternotomy, weighted, placed in ice-cold modified Krebs-Henseleit (KH) solution (in mmol/L): NaCl (118.0), KCl (4.7), CaCl_2_ (2.5), KH_2_PO_4_ (1.2), Mg_2_SO_4_ (1.2), Na- pyruvate (2.0), NaHCO_3_ (25), and glucose (11.0) at pH 7.4–7.5 and mounted on the perfusion apparatus (model IH-SR, Hugo Sachs Elektronik-Harvard Apparatus GmbH, Germany) with the time delay <4 min. In order to facilitate coronary effluent drainage, a small incision was made at the root of the pulmonary artery. Perfusion of the heart was performed in the Langendorff mode and at constant pressure of 80 mmHg with continuously warmed (37°C), aerated (95% O_2_ with 5% CO_2_) and filtered (in-line 0.45 µm Sterivex-HV filter) KH solution. Throughout the experiment, the heart was kept in the enclosed water-jacketed chamber with ambient temperature maintained at 37°C±0.7 as monitored by a laboratory mercury thermometer. Temperature of the perfusion solution (37±0.3°C) was continuously measured by the thermocouple microprobe placed at the entry into the aortic cannula. Coronary flow rate was recorded via an ultrasonic flow meter probe integrated in the aortic block placed above the heart. The heart was allowed to beat spontaneously and its electrical activity was monitored by epicardial ECG recording obtained via two Ag/AgCl electrodes positioned on the right atrium and the apex. Aortic pressure, coronary flow, ECG and perfusate temperature were continuously recorded using the 16-channel Powerlab system (ADInstruments, Oxford, UK) and LabChart 7 software. Hearts were randomized into the control (CTRL) group and ischemic preconditioning (IP) group. CTRL group was subjected to 45 min equilibration followed by 33 min global zero-flow ischemia and 60 min reperfusion. Experimental animals in the IP group were subjected to 25 min equilibration period then to 2 cycles of 5 min global zero-flow ischemia and 5 min reperfusion, followed by 33 min ischemia and 60 min reperfusion. Seven hearts were excluded from the study as they did not fulfill one of the following inclusion criteria: (i) time delay to cannulation <4 min, (ii) basal coronary flow <5 mL/min (indicating absence of aortic damages), (iii) no sustained arrhythmia and heart rate >300 bpm at the end of equilibration period.

### Measurements of infarct area

After the completion of reperfusion, hearts were immediately frozen at −80°C for 10 minutes and then cut into 5–6 transverse slices and stained in 1% TTC (2,3,5-triphenyl-tetrazolium chloride in phosphate buffer (88 mmol/l Na_2_HPO_4_, 1.8 mmol/l NaH_2_PO_4_, pH = 7.4) for 15 min in 37°C. TTC was then replaced with 4% formaline for at least 16 h before each individual slice was blotted, weighted and scanned at the resolution of 1200 dpi (Canon scanner, LIDE70, Denmark). Determination of infarct size was performed as previously described [26]. Briefly, the total areas of individual slices, areas at risk (AAR_n_), and areas of infarction (AI_n_) were measured by planimetry (Image J 2.0 software, NIH, USA) and infarct size was calculated as % of total cardiac mass (AAR) and determined as the weighted mean of individual slice values: (AI_n_/AAR_n_)×(W_n_/W_total_), where W_n_ is the weight of the respective section (n) and W_total_ is the sum weight of all slices. Quantification of infarct size was performed in a blinded manner in respect to genotype and experimental protocol. Hearts from both sex were pooled since their susceptibility to the ischemic insult per se did not differ and they showed an equal responses to the IP (gender-related data not shown).

### Statistics

**Figure 1:**
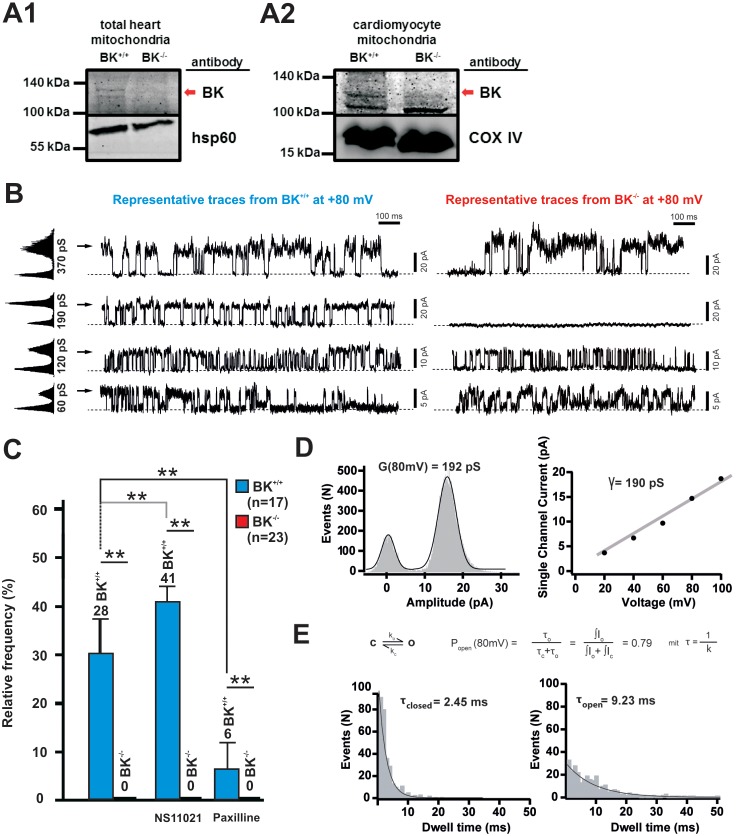
Paxilline and NS11021 sensitive mitoBK channels give rise to a 190 pS conductance, which is absent in BK-negative mitoplasts. (**A1+2**) Expression analysis of BK channels mitochondrial protein fractions of adult heart (A1) and cardiac myocytes (A2) from BK^−/−^ and litter-mate BK^+/+^ mice. Immune-reactive bands corresponding to the predicted molecular weight (approx. 120 kDa) of BK channels were detected only in mitochondrial protein fractions derived from BK^+/+^ hearts and cardiac myocytes (red arrow), whereas mitochondrial lysates from BK^−/−^ tissues remained BK-negative. By identifying cytochrome c oxidase IV (COX IV) or heat shock protein 60 (hsp60) equal loading of the samples was confirmed. A fluorescent protein marker was used to estimate the sizes of the respective proteins (not shown). (**B**) Four different conductances were observed in mitoplasts from cardiomyocytes in a voltage step protocol with 2 seconds at +80 mV followed by 3 seconds at holding potential (−50 mV). Internal to external potassium ratio was 130∶10 mmol/l during the experiment. The magnitude of the occurring conductances under these conditions was calculated to approx. 370, 190, 120 and 60 pS. The figure displays individual representative traces of different patches which show a specific activity at the recorded time. Arrows indicate open state. (**C**) The relative frequency over 3 minutes of recording of the 190 pS (resembling BK K^+^-currents) conductance decreased from 29%±8% in BK^+/+^ mice to 0% in BK^−/−^ mice. NS11021 increased the incidence of the 190 pS conductance to 41±3%, whereas the BK opener had no effect on BK-negative mitoplasts and paxilline reduced the incidence to 6±5% in BK^+/+^ mitoplasts (number of single measurements was n = 17 for BK^+/+^ and n = 23 for BK^−/−^, number of mitoplasts preparations was four for each group). Statistical analyses were performed on BK^+/+^ mitoplasts recorded in the untreated (basal) versus treated states (paxilline or NS11021). Additionally, all three conditions (basal, paxilline, NS11021) were tested for significant differences between the two genotypes (*P<0.05, **P<0.01). (**D**) Single channel analysis of an individual trace of a 190 pS conductance. The histogram with Gaussian fit results in a conductance of 192 pS. Additionally, slope conductance was determined by linear fit of current amplitude *versus* voltage confirming a conductance of 190 pS. (**E**) When assuming an easy two state model open probability can be calculated by idealization of channel openings and analysis of dwell times. This results in an open probability of 0.79 at 80 mV.

**Figure 2:**
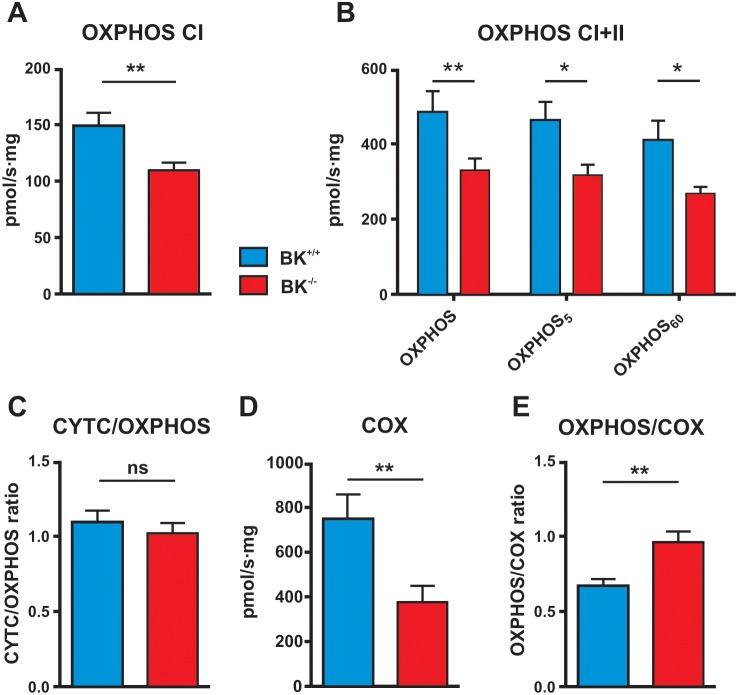
Mitochondrial respiratory responses in mouse left heart ventricular fibres. O_2_-consumption of permeabilized BK^+/+^ fibres (blue bars) and fibres isolated from BK^−/−^ hearts (red bars) were measured by applying an *in vitro* model of I/R at normoxia and reo-xygenation (both at 21% O_2_) upon 60 min of anoxia (by a N_2_ gassing system). For a representative original recording trace please refer to Figure S2 in File S1. (**A**) Coupled state 3 respiratory rate (OXPHOS) with complex I (CI) substrates malate (2 mmol/l) and glutamate (10 mmol/l) in the presence of ADP (5 mmol/l). (**B**) Cumulative OXPHOS of CI and II upon subsequent addition of succinate (10 mmol/l) to (A) for a supply of electrons to complex II (CII). OXPHOS of CI and II after 5 min (OXPHOS_5_) and after 60 min (OXPHOS_60_) of anoxia are shown. (**C**) Ratio of respiration upon addition of cytochrome c (CYTC) 5 min after 60 min anoxia compared to steady state respiratory flux rate before anoxia as a test for intactness of the IMM. (**D**) Isolated activity of complex IV (cytochrome c oxidase (COX)) with redox substrates ascorbate (2 mmol/l) and TMPD (5 mmol/l) in the presence of complex III blocker Antimycin A (2 µmol/l). (**E**) Ratio of OXPHOS to COX activity under fully oxygenated conditions showing loss of COX excess capacity in BK^−/−^ as the ratio approaches 1.0 revealing an insufficient reserve capacity to produce energy in mitoBK-deficient fibres. Data are mean ± SEM with n = 6 for BK^+/+^ and n = 9 for BK^−/−^ with ns = non-significant and **P<0.01 significantly different compared to BK^+/+^ (Fig. 2A, C, D two-tailed paired t-test). *P<0.05, **P<0.01, significantly different compared to BK^+/+^ within the respective condition (Fig. 2B).

**Figure 3:**
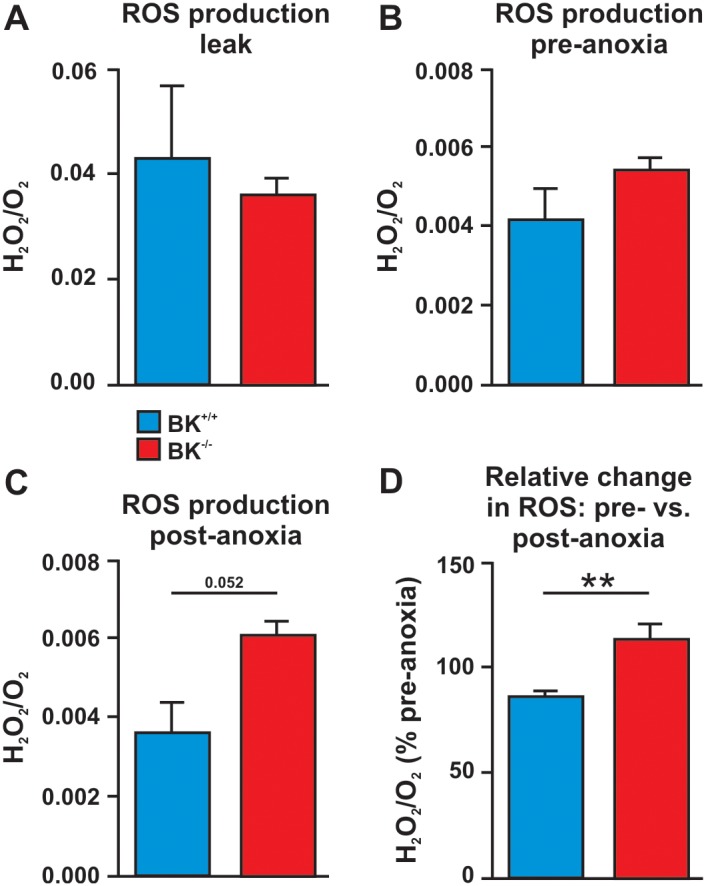
Simultaneous ROS production and respiration measurements of isolated BK^+/+^ and BK^−/−^ mitochondria. Oxygen consumption and ROS are determined by using an oxygraph with fluorometric detection of H_2_O_2_ production as H_2_O_2_ production expressed relative to oxygen consumption (pmol•pmol^−1^). (**A**) “Leak” is the respiratory state were cardiomyocyte mitochondria are exposed to the substrates pyruvate (5 mM), malate (2 mM) and succinate (10 mM) without ADP. The resulting increase in substrate-induced membrane potential enhanced ROS production of BK^+/+^ and BK^−/−^ mitochondria to a similar extent. (**B**) Respiration was stimulated by ADP (2 mM) and oxygen pressure was lowered from ∼21.2 kPa to ∼3 kPa. ROS production at peak respiration rate before O_2_ limits respiration (prior to the O_2_-dependent hyperbolic decline in respiration that occurs below 3 kPa) was termed “preanoxia”, and again there was no difference between BK^+/+^ and BK^−/−^ mitochondria. (**C**) After 20 minutes of complete anoxia, the chambers were re-oxygenated to ∼3 kPa and ROS production was quantified as “postanoxia”. (**D**) Post-anoxic related to pre-anoxic ROS production of BK^+/+^ mitochondria was significantly lower than in BK^−/−^ mitochondria. Data are mean ± SEM, n = 3 to 4 per experiment and genotype. **P<0.01 significantly different compared to BK^+/+^ (two-tailed paired t-test).

**Figure 4:**
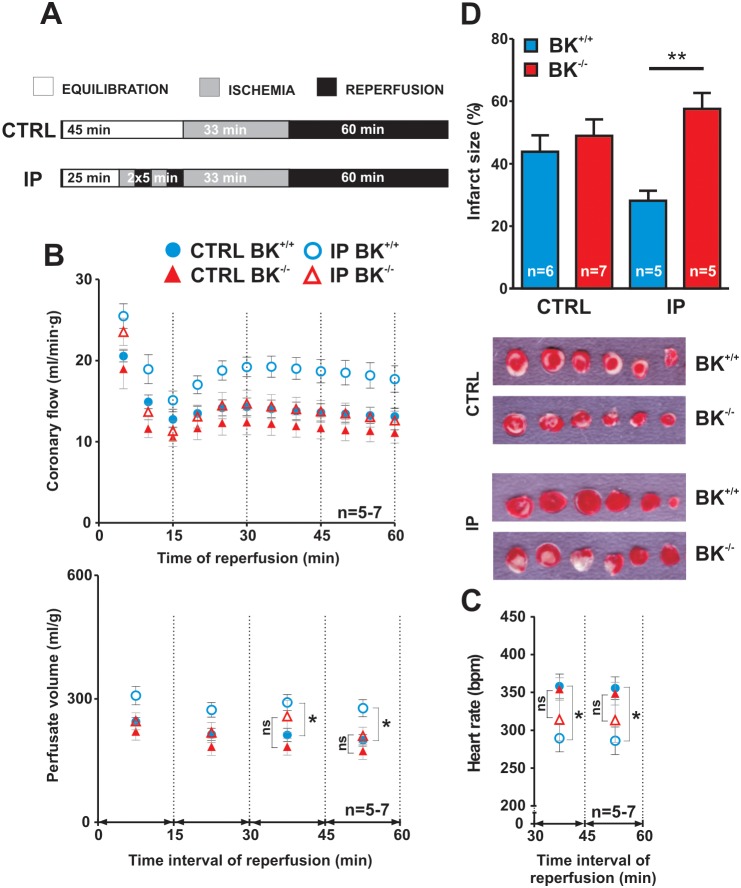
Loss of myocardial ischemic preconditioning in BK^−/−^ hearts. (**A**) Schematic illustration of the protocols used for the assessment of I/R injury in isolated, perfused hearts of BK^+/+^ and BK^−/−^ mice. CTRL indicates a control protocol with 33 min global zero-flow ischemia followed by 60 min reperfusion and IP denotes an ischemic preconditioning protocol consisting of 2 extra cycles of 5 min ischemia and 5 min reperfusion preceding the global ischemic insult. (**B**) Effects of CTRL and IP protocol on post-ischemic coronary flow rate normalized to heart weight during reperfusion (upper), with each point depicting mean values ± SEM of the preceding 5 min recording. Coronary flow is significantly different as tested by Two-way ANOVA. Lower panel shows that IP improves coronary flow in the BK^+/+^ but not in the BK^−/−^ group as evidenced by increased perfusate volume at reperfusion (area under the curve from the upper panel) at the time interval from 30 to 45 min and from 45 min to 60 min (ns = non-significant, *P<0.05 for CTRL BK^+/+^ *versus* IP BK^+/+^; Two-way ANOVA followed by a Bonferroni post-hoc test). (**C**) Effects of CTRL and IP protocol on post-ischemic heart rate in BK^+/+^ and BK^−/−^ group. Each point depicts mean values ± SEM of the 15 min interval recording excluding rare extrasystolic beats. Due to multiple sustained arrhythmias in the first 30 min of reperfusion, heart rate could not be reliably measured at the interval from 0–30 min (no differences between genotypes). Heart rate of BK^+/+^ hearts is lower in the IP than in the CTRL group (*P<0.05 CTRL BK^+/+^ *versus* IP BK^+/+^; Two-way ANOVA followed by a Bonferroni post-hoc test). (**D**) Effects of CTRL and IP protocol on infarct size in BK^+/+^ and BK^−/−^ groups (upper panel). Infarct size is comparable between BK^+/+^ and BK^−/−^ hearts in the CTRL group, but IP failed to protect BK^−/−^ hearts resulting in smaller infarct sizes of BK^+/+^ hearts (**P<0.01; One-way ANOVA followed by Tukey’s multiple comparison test). All values are means ± SEM. Data from male and female BK^+/+^ and accordingly BK^−/−^ hearts were pooled since sub-group analyses revealed that differences in the infarct sizes did not depend on sex. Lower panels demonstrate representative images of myocardial sections stained with tetrazolium chloride where red color indicates viable tissue and un-stained pale color demarcates infarcted areas.

Data are expressed as mean ± SEM. Electrophysiological results of BK^+/+^ were tested by a one-way ANOVA, and compared to BK^−/−^ by a two-way ANOVA (Fig. 1). Electrophysiological experiments were treated as Bernoulli experiments. The effect of genotype on respiration and ROS production was evaluated using un-paired t-test (Fig. 2, Fig. 3 and Figure S3 in File S1) and a Two-way analysis of variance (ANOVA) followed by a Tukey’s multiple comparison test (Fig. 2B). Infarct size was compared using One-way analysis of variance (ANOVA) test followed by a Turkey’s multiple comparison test (Fig. 4B). Morphometric characteristic and basal coronary flow and heart rate in 4 experimental groups was analyzed using One-way ANOVA test (Table S1 in File S1). Effects of the experimental protocols and genotypes on post-ischemic coronary flow and heart rate were compared using Two-way ANOVA followed by Bonferroni post-hoc test (Fig. 4C and D). Total reactive hyperemic volume was compared using an un-paired t-test (Figure S4 in File S1). Errors estimate was done by calculating the variability across different animals. Probability value of P<0.05 was considered significant.

## Results

We first confirmed that the BK channel is detectable in mitochondrial protein fractions isolated from BK wild-type (BK^+/+^) hearts and CMs, whereas mitochondrial fractions derived from the respective BK^−/−^ cells/organs remained BK negative (Fig. 1A). As compared to control tissues (e.g. cerebellum (not shown)) BK protein expression levels in heart and CM mitochondria were very low, however, the molecular weight of the immuno-positive bands indicate that mitoBK channels have a similar molecular identity of approx. 120 kDa as canonical BKs usually present in the plasma membrane of cells. To examine the electrophysiological and pharmacological properties of the mitoBK we studied mitoplasts, generated by osmotic swelling. The quality of these purifications i.e. the strongly availability of contaminant mitochondrial outer membranes was confirmed by high magnification microscopy (Figure S1A in File S1). Under the given experimental conditions (s. Methods) we observed ionic conductances of 4 magnitudes in BK^+/+^ mitoplasts with a different relative frequency during 9 minutes of recording: ∼60 pS (35%), ∼120 pS (80%), ∼190 pS (55%) and ∼370 pS (15%) (Fig. 1B). Importantly, only the ∼190 pS conductance was absent from BK^−/−^ mitoplasts, whereas all other magnitudes were still present. Therefore, subsequent analyses focused on the ∼190 pS conductance. We separated the patch-clamp recordings into 3 identical intervals of 3 min duration. After the first control phase resembling 3 min of baseline measurement, NS11021 (10 µmol/l) was applied with the external solution, which was exchanged by paxilline (100 nmol/l in external solution) 3 min later (Fig. 1C). NS11021 increased the relative frequency of the ∼190 pS conductance by 12%, whereas paxilline superfusion had the opposite effect reducing the relative frequency of the respective conductance to 6% in BK^+/+^ mitoplasts. In BK negative mitochondria neither NS11021 nor paxilline had an effect strongly supporting the notion that mitoBK channels are the product of the KCNMA1 gene targeted by our global BK channel knockout strategy [36]. For a better characterization of the mitoBK we examined phases of single channel activity. These in-depth analyses of representative traces revealed a slope conductance of 190 pS and an open probability of 0.79 at +80 mV for the murine mitoBK (Fig. 1D and E). Importantly, these data complement very recent findings by Singh and co-workers [47], and together provide strong evidence for mitoBK channels encoded by the murine KCNMA1 gene in cardiac myocytes.

We next performed high-resolution respirometry measurements on permeabilized myocardial muscle fibres to test whether the ablation of mitoBK has any consequences for mitochondrial function at normoxia or anoxia with re-oxygenation. A representative trace is shown in Figure S2 in File S1. Mitochondrial OXPHOS capacity for the complex I (CI) substrate glutamate was reduced in BK^−/−^ (Fig. 2A) compared to BK^+/+^ fibres (109±7 vs. 149±12 pmol⋅sec^−1^⋅m g^−1^, P<0.05), as was the maximal mitochondrial flux rate with convergent electron supply through CI+II (329±35 vs. 483±57 pmol⋅sec^−1^⋅mg^−1^, P<0.05, Fig. 2B). Accordingly, the substrate control ratio (CI/CI+II) was similar for BK^−/−^ (0.35±0.01) and BK^+/+^ (0.34±0.03) indicating a proportional down-regulation of coupled respiration of the electron transfer system (ETS). Next, we tested the response of the fibre preparations to different levels of oxygenation *in vitro*. Following the 5 min period of anoxia, mitochondrial respiration rate with re-oxygenation recovered in both BK^+/+^ (from 483±57 to 462±51 pmol⋅sec^−1^⋅mg^−1^) and BK^−/−^ (329±35 to 316±31 pmol⋅sec^−1^⋅mg^−1^, Fig. 2B) fibres to almost the same level as the pre-anoxia level. Following a subsequent period of 60 min of anoxia mitochondrial respiration was 15% lower in BK^+/+^ and 19% in BK^−/−^ fibres. Although this did not reach significance respiration rates remained lower in BK^−/−^ compared to BK^+/+^ following 5 and 60 min of anoxia (p<0.05, Fig. 2B) in BK^+/+^ and BK^−/−^ fibres as compared to pre-anoxia levels, respectively. A decline in the OXPHOS recovery is indicative of either anoxic fragility, disruption of the membrane potential and/or diminished activity of the IMM respiratory complexes. Since no additive responses to subsequent addition of external cytochrome c were detected in either groups (Fig. 2C) we conclude that the OMM remained intact following the anoxic stimuli. However, the isolated respiratory flux rate of cytochrome c oxidase (COX) [48] (747±99 pmol⋅sec^−1^⋅mg^−1^, P<0.05, Fig. 2D) was higher than total OXPHOS capacity in BK^+/+^ fibres (OXPHOS to COX ratio 0.66±0.04, Fig. 2E) observed under normoxic conditions, whereas in the BK^−/−^ no excess capacity of COX (375±66 pmol⋅sec^−1^⋅mg^−1^, P = 0.48, Fig. 2D) over OXPHOS (OXPHOS to COX ratio 0.96±0.06, Fig. 2E) could be observed indicating a loss of the bioenergetic reserve of respiration coupled to ATP production. As an ultimate proof that the changes observed in mitochondrial functions resulted from a distorted activity of the respiratory chain enzymes upon mitoBK ablation and not due to structural changes of the CM mitochondriome, we performed transmission electron microscopy (TEM) analyses of BK^+/+^ and BK^−/−^ ventricle fibres (Figure S1B in File S1). TEM [49] images from mitochondria in the rested, unstressed state indicate neither apparent down-regulation of mitochondrial volume nor distinct morphological changes across multiple sections. Indeed, the TEM revealed a highly dense distribution of mitochondria in fibres from both genotypes, evidence of mega-mitochondria, fusion and a vast network of interfibrillar and subsarcollemal mitochondria with no signs of cristae disruption, or inter-membrane swelling (Figure S1B in File S1). Extending from these observations the functional marker of a higher OXPHOS to COX respiration ratio in mitoBK-deficient fibres (0.9 *vs*. 0.65) indicates dysregulated mitochondrial function at normoxia and re-oxygenation linked to a loss of excess capacity of COX i.e. independent of structural deficits such as density or volume of the mitochondria (Figure S1B in File S1). Since OXPHOS capacities are to some extent diminished in both BK^+/+^ and BK^−/−^ fibres upon anoxia (Fig. 2B), we conclude that the BK ablation i.e. absence of mitoBK *per se* does not affect the OXPHOS response to a limited supply with O_2_. Further, our *in vitro* O_2_-respiration experiments may imply that protective factors need to be released (possibly outside the CM) to signal to the CM mitochondrion for establishing mitoBK-dependent cardio-protection. To test whether this signalling model applies to all types of striated muscles or if the observed effects are specific for the myocardium we performed a related set of respirometry measurements on BK^+/+^ and BK^−/−^ soleus muscle fibre bundles (Figure S3 in File S1). Collectively, these analyses reveal no differences between the two genotypes indicating that mitochondrial bioenergetics upon BK ablation are only affecting the heart.

In order to mechanistically substantiate the role of the endogenous mitoBK we next recorded OXPHOS and ROS production simultaneously from isolated cardiac mitochondria. After 20 minutes of complete anoxia ROS production (normalized to OXPHOS capacity) at fully oxygenated conditions or upon deprivation of ADP were not different between BK^+/+^ and BK^−/−^ mitochondria (Fig. 3A and B). However, post-anoxic ROS generation rates from BK^−/−^ mitochondria were elevated (Fig. 3C) and this difference reached significance when comparing pre-/post-anoxic ROS/OXPHOS ratios of both genotypes (Fig. 3D).

Since elevated ROS at re-oxygenation and a loss of bioenergetic reserves for coupled respiration and ATP production renders CMs less capable of generating sufficient energy supply for contractile and homeostatic function under high loading conditions or ischemia we addressed the role of cardiac BK channels for cardio-protection in the well-established isolated, beating heart model [26].

In a first series of experiments, baseline responses of isolated hearts derived from BK^+/+^ and BK^−/−^ mice to I/R (CTRL) were analyzed (Fig. 4A and Table S1 in File S1). Infarct sizes of BK^+/+^ and BK^−/−^ hearts subjected to 33 min ischemia and 60 min reperfusion were not different. Similarly, coronary flow rates, perfusate volume and heart rates (Fig. 4B and C) measured at reperfusion were similar between both genotypes indicating that mitoBK channels *per se* do not affect myocardial survival or function upon a prolonged ischemic insult. In the next series of experiments, BK^+/+^ and BK^−/−^ hearts were primed with two, transient periods of ischemia and reperfusion prior to the prolonged ischemia (Fig. 4A). Infarct size at I/R was significantly lower in BK^+/+^ hearts as compared to infarct size in BK^−/−^ hearts upon IP (IP BK^+/+^ 28±3% versus IP BK^−/−^ 58±5% infarct size of the area at risk (AAR), n = 5, **P<0.01) (Fig. 4D). Interestingly, BK ablation fully abrogated beneficial effects of the IP supporting the presented molecular, electrophysiological, pharmacological (Fig. 1A–D) and biochemical conclusions (Fig. 2 and 3) that KCNMA1 encoded cardiac BK channels i.e. mitoBKs are targeted by survival pathways at I/R injury [47]. In addition to differences in infarct sizes, coronary flow at reperfusion was improved by IP in the BK^+/+^ hearts, but not in BK^−/−^ hearts (Fig. 4B). In particular, the perfusate volume used by the BK^+/+^ hearts towards the end of reperfusion, in two time intervals from 30 to 45 min and from 45 to 60 min, was significantly higher in cardiac preparation subjected to IP. Several studies did observe an increase of the post-ischemic coronary flow upon protective IP associated with smaller infarct size [26], [50].

Taken together, our data from the isolated perfused heart model shows that cardio-protective effects of IP depend on cardiac BK channel.

## Discussion

### mitoBK channels are the product of the KCNMA1 gene in cardiomyocytes

Since the first reports on cardio-protective “mitoBK channel-like proteins” [15], [51] it has been questioned whether mitoBKs are indeed a product of the KCNMA1 gene, and if they share identical features with canonical BK channels usually found at the plasma membrane of SMCs, neurons and endocrine cells [52]. We support very recent findings [47] showing that an approx. 120 kDa protein in the IMM is identical to the mitoBK channel derived from KCNMA1 in CMs. Singh et al. [47] detected several splice-variants and an insertless BK mRNA in murine CMs. By an elegant set of experiments the authors further confirmed that only the most abundant CM BK variant DEC with a C-terminal extension of 50-aa (named after the last 3 aa of the insert), was targeted efficiently to mitochondria [47]. Herein, we add several new aspects: 1.) mitoBK is a paxilline- and NS11021-sensitive BK channel with an open-probability of 0.79 at +80 mV and a slope conductance of approximately 190 pS, 2.) mitoBKs are important for full capacity of OXPHOS and the ETS under oxygenated and anoxic conditions, 3.) lack of mitoBK stimulates ROS production upon anoxia with subsequent re-oxygenation, and 4.) protection afforded by IP (in addition to NS1619 [47]) requires the mitoBK.

### Altered oxidative energy supply and ROS production in BK-negative permeabilized muscle fibres and cardiomyocyte mitochondria

The mitochondrial O_2_-respirometry experiments performed on BK^−/−^ heart muscle fibres reveal that OXPHOS capacity with substrate supply to both CI and II of the ETS were proportionately attenuated (Fig. 2). The similar substrate control ratio (BK^−/−^ (0.35±0.01) and BK^+/+^ (0.34±0.03) for CI/CI+CII) indicates a functional deficit in maximal cellular oxidative energy supply. Convergent electron input to CI and II (Fig. 2B), which is the physiologically relevant maximal coupled mitochondrial respiration, elicited higher respiratory values as compared to the isolated respiration of CI in both genotypes (Fig. 2A). Interestingly, brief and prolonged periods of anoxia interfered with the recovery of the OXPHOS capacity upon re-oxygenation in BK^+/+^ and BK^−/−^ fibres (Fig. 2B). We are aware of the limitations of this *ex-vivo* system because it is unlikely that a single episode of 5 min anoxia *ex-vivo* (prior to the prolonged anoxia) adequately elicits IP triggered cardio-protective pathways as observed in full heart preparations i.e. those factors that signal to CM mitoBKs (or vascular BKs). The loss of IP in BK^−/−^ hearts does not necessarily stem from the decline in the OXPHOS recovery responses, because it was observed in BK^+/+^ and BK^−/−^ fibres after the initial 5 min anoxia period. Moreover, our combined ROS and respirometry measurements (Fig. 3) indicate a mitoBK-dependent control over mitochondrial ROS generation at re-oxygenation, whereas ROS production at pre-anoxia (Fig. 3A and B) was not affected by endogenous lack of BK. Because the co-administration of ROS scavengers has been shown earlier to attenuate pre-conditioning-like effects afforded by BK-openers such as NS1619 [53], we conclude that BK at the IMM plays a dual role by fine-tuning protective and toxic ROS under limited O_2_ conditions and conditions of O_2_ reintroduction thereby integrating to survival pathways at I/R. However, neither anoxic episodes *ex-vivo*, nor the amount of I/R injury revealed any differences between the two genotypes with respect to the anoxia-induced decrease in OXPHOS capacities (Fig. 2B) or infarct size (Fig. 4D). Thus, differences in the experimental setups of IP at the beating heart model and anoxia in isolated mitochondria may contribute to control of mitochondrial ROS formation. It is noteworthy that mitochondria *ex-vivo* are remarkably robust, being able to tolerate a prolonged period of anoxia, with recovery of OXPHOS capacity to ∼80% of pre-anoxia levels (Fig. 2B).

### Cardiac BK channels contribute to the protective signalling afforded by ischemic preconditioning while lack of mitoBKs causes a functional impairment of the respiratory chain

BK^+/+^ and BK^−/−^ mitochondria are intact with respect to TEM ultra-structures (Figure S1B in File S1), recovery responses from anoxia (Fig. 2B) and the lack of a cytochrome c response (Fig. 2C). The extent of ischemic infarction (in the absence of IP) in the Langendorff-perfused heart stems from ischemic signalling events within the myocardium independent of endogenous mitoBK activity. This is in good agreement with other studies of I/R injuries in intact heart preparations at baseline [34], [35]. However, we and others [47] find mitoBK channels are involved in the IP-mediated cardio-protection.

This is in accordance with earlier pharmacological studies [28] with BK channel activators (NS1619, NS11021) applied prior to ischemia exhibiting significant cardio-protective effects at I/R [15], [26]. Pharmacological activation of the channel depolarizes the IMM potential and attenuates mitochondrial Ca^2+^ accumulation [16], thereby, the mitoBK channels could serve a similar role as in the plasma-membrane of electrically excitable cells where BK prevents Ca^2+^ accumulation. Furthermore, opening of mitoBK channels has been reported to mildly increase ROS production during preconditioning, with the formed ROS acting as an important signaling molecule for triggering pharmacological preconditioning [18], [53]. Similarly, with regards to IP, ROS formed at IP acts as an important trigger of cardio-protection [54], [55]. Finally, studies have indicated that activating K^+^-flux through mitoBKs improves the energetic performance of cardiac mitochondria, fine-tuning the mitochondrial volume to optimize oxidative phosphorylation [56]. These findings support our observations of a reduced functional capacity of OXPHOS and loss of cardio-protection by IP in BK^−/−^ hearts. Consistent with the impaired OXPHOS responses of CI and CI+II under fully oxygenated conditions, the isolated activity of COX was also markedly reduced in the BK^−/−^ ventricular fibres. This indicates that the absence of the mitoBK channels causes a functional impairment of sequential components of the respiratory chain as previously reported [23]. Under physiological conditions COX excess capacity maintains a high affinity for O_2_ during coupled respiration, a low membrane potential, a corresponding low PO_2_ at cytochrome aa3 and thus a high PO_2_ gradient from the capillary to mitochondria facilitating O_2_ diffusion [57]. The finding that OXPHOS operates at near maximum COX activity in BK^−/−^ suggests a more reduced redox state of COX during ADP-stimulation respiration [58], [59]. In this setting, a higher ROS production has been reported [59], [60], which may alter diverse signalling pathways and functional characteristics of mitochondria [57], [61] and indeed we find elevated ROS release from BK^−/−^ mitochondria only upon O_2_ reintroduction after prolonged anoxia *in vitro*.

The finding that a prolonged period of 60 min of anoxia significantly (and equally) reduced OXPHOS recovery in both genotypes, but to different absolute levels, further supports the notion of a loss of key regulatory signals in the BK^−/−^ hearts during the IP experiments and points to the different infarct size between the groups. The functional marker of a higher OXPHOS:COX respiration ratio in the BK^−/−^ fibres (0.9 vs. 0.65 BK^+/+^) indicates a dysregulation of mitochondrial functions linked to loss of excess capacity of COX independent of density or volume of the mitochondria. Both Ca^2+^ and ROS may activate the BK channel [18], which is thought to control Ca^2+^ and ROS levels in mitochondria upon limited O_2_ or O_2_ re-introduction, conferring cardio-protection against cellular stress, mitochondria permeability pore opening and Bax-mediated pro-apoptotic events [62]. The combined responses of reduced OXPHOS capacity, lower COX activity and elevated ROS production from BK^−/−^ mitochondria suggest a mechanism for loss of cardio-protection by IP in BK^−/−^ mice.

### Limitations of the presented study

Obviously, our report does not exclude the possibility that the protection afforded by the IP protocol is also influenced by vascular BK channels. Differences in infarct size and the parallel perfusion response following pre-conditioning (Fig. 4B and D) might indeed reflect a close matching between convective O_2_ delivery and metabolic demand of the hearts. It is possible that the differences in perfusion responses between the two genotypes following IP reflect differences in the mass of viable cells and subsequently a reduced corresponding O_2_ demand in the BK^−/−^. Additionally, the metabolic demands of the BK-negative hearts could be lower due to differences in heart rates in the Langendorff-perfused heart model (Fig. 4C). BK channel activity in coronary smooth muscle cells (SMCs) or elsewhere could contribute to vasodilation in BK^+/+^ and consequently to changes in hemodynamics. Alternatively, the ischemia may induce overlapping vaso-regulatory signals that may be independent of the O_2_ demand of the myocardium. Direct vascular effects due to opening of SMC BK channel upon reperfusion are unlikely since BK^−/−^ hearts show a strongly preserved reactive hyperemic response upon a transient 5 min ischemia, an uncompromised baseline coronary flow (Figure S4 in File S1), and the improvement in myocardial functions observed with the BK openers NS1619 [47] or NS11021 were independent of significant increases in flow [26].

Conditional and inducible KCNMA1 mutant mouse models lacking the BK exclusively in SMCs, platelets, intrinsic cardiac neurons or cardiomyocytes are urgently needed to discriminate these details *in vivo* [35], [63], [64].

## Supporting Information

File S1 **Figure S1,** Imaging of isolated mitoplasts and ventricular mitochondria. **Figure S2,** Original traces of high-resolution respirometry recorded in permeabilized BK^+/+^ and BK^−/−^ heart muscles. **Figure S3,** Mitochondrial respiratory responses in BK^+/+^ and BK^−/−^ mouse skeletal muscle fibres. **Figure S4,** Hemodynamics of BK^+/+^ and BK^−/−^ hearts upon 5 min of ischemia and 5 min of reperfusion. **Table S1,** Summary of parameters assessed in BK^+/+^ and BK^−/−^ hearts subjected to the *ex-vivo* Langendorff preparation.
(DOC)
