# Supplementary material for: KCNMA1 Encoded Cardiac BK Channels Afford Protection against Ischemia-Reperfusion Injury
Source: PLoS One. 2014 Jul 29;9(7):e103402. doi: 10.1371/journal.pone.0103402 (PMC4114839; doi:10.1371/journal.pone.0103402)
Supplement: File S1 — Figure S1, Imaging of isolated mitoplasts and ventricular mitochondria. Figure S2, Original traces of high-resolution respirometry recorded in permeabilized BK+/+ and BK−/− heart muscles. Figure S3, Mitochondrial respiratory responses in BK+/+ and BK−/− mouse skeletal muscle fibres. Figure S4, Hemodynamics of BK+/+ and BK−/− hearts upon 5 min of ischemia and 5 min of reperfusion. Table S1, Summary of parameters assessed in BK+/+ and BK−/− hearts subjected to the ex-vivo Langendorff preparation. (DOC) [file pone.0103402.s001.doc]

**KCNMA1 encoded cardiac BK channels afford protection against ischemia-reperfusion injury**

Ewa Soltysinska^1,2^, Bo Hjorth Bentzen^1^, Maria Barthmes^3,4^, Helle Hattel^2^, A. Brianne Thrush^5^, Mary-Ellen Harper^5^, Klaus Qvortrup^6^, Filip J. Larsen^7^, Tomas A. Schiffer^7^, Jose Losa-Reyna^7^, Julia Straubinger^8^, Angelina Kniess^8^, Morten Bækgaard Thomsen^1,2^, Andrea Brüggemann^4^, Stefanie Fenske^3^, Martin Biel^3^, Peter Ruth^8^, Christian Wahl-Schott^3^, Robert Christopher Boushel^2,*^, Søren-Peter Olesen^1,2, *,§^, Robert Lukowski^8,*,§^

^1^The Danish National Research Foundation Centre for Cardiac Arrhythmia, University of Copenhagen, Copenhagen, Denmark

^2^Department of Biomedical Sciences, Faculty of Health and Medical Sciences, University of Copenhagen, Copenhagen, Denmark

^3^Center for Integrated Protein Science Munich (CIPSM), Ludwig-Maximilians-Universität, Munich, Germany; Department of Pharmacy, Center for Drug Research, Ludwig-Maximilians-Universität, Munich, Germany

^4^Nanion Technologies GmbH, Munich, Germany

^5^Department of Biochemistry, Microbiology and Immunology, Faculty of Medicine, University of Ottawa, Ottawa, Canada

^6^Department of Biomedical Sciences, Core Facility for Integrated Microscopy, Faculty of Health and Medical Sciences, University of Copenhagen, Copenhagen, Denmark

^7^Department of Physiology and Pharmacology, Karolinska Institutet, Stockholm, Sweden

^8^Department of Pharmacology, Toxicology and Clinical Pharmacy, Institute of Pharmacy, University of Tübingen, Tübingen, Germany

*These authors contributed equally to the presented work

^§^Corresponding authors:

Søren-Peter Olesen

The Danish National Research Foundation Centre for Cardiac Arrhythmia

Department of Biomedical Sciences

The Panum Institute

University of Copenhagen

Tel. +45 20 28 97 06

Fax +45 35 32 75 55

E-mail: [spfo@sund.ku.dk](mailto:spfo@sund.ku.dk)

Robert Lukowski

Department of Pharmacology, Toxicology and Clinical Pharmacy

Institute of Pharmacy

University of Tübingen

Tel. +49 7071 2974550

Fax +49 7071 292476

E-mail: [robert.lukowski@uni-tuebingen.de](mailto:robert.lukowski@uni-tuebingen.de)

Supporting information: Supporting Figures and Legends: (4)

Supporting Tables and Legends: (1)

**Supporting** **Information** **Legends to Figures and Tables**

**Figure S1: Imaging of isolated mitoplasts and ventricular mitochondria**

**(A)** Optical imaging of the cardiac mitoplast fraction. White arrows indicate intact mitoplasts. The inlay reveals that upon osmotic swelling only small remnants of the OMM (arrowhead) are still attached to the unfolded IMM, which thereby forms considerably enlarged spherical mitoplasts. **(B)** Transmission electron micrographs of left ventricular myocytes from BK^+/+^ (left panels) and BK^-/-^ (right panels). (M) mitochondrium, (C) capillary. Scale bars top: 2 µm. Scale bars bottom: 500 nm.

**Figure S2: Original traces of high-resolution respirometry recorded in permeabilized BK^+/+^ and BK^-/-^ heart muscles.**

O_2_ concentration (blue tracing, corresponding to left Y-axis) and respiration rates (red tracing, corresponding to right Y-axis) are shown for representative muscle fibres. Substrate and co-factor supply to the fibres and oxygenation (see below) are indicated. Abbreviations used are: 10 mmol/l glutamate (g), 5 mmol/l ADP (adp), 10 mmol/l succinate (s), 5 min of anoxia (anoxia5), 60 min of anoxia (anoxia60), 10 μmol/l cyctochrom c (cytc), 2.5 μmol/l Antimycin A (aa), 5 mmol/l TMPD and 2 mmol/l ascorbate (t+a), 100 mmol/l sodium azide (a+z). Intervals highlighted in grey were used for the determination of time-averaged O_2_ flux rates (consult Fig. 2).

**Figure S3: Mitochondrial respiratory responses in BK^+/+^ and BK^-/-^ mouse skeletal muscle fibres**

O_2_-consumption of permeabilized soleus muscle fibres isolated from BK^+/+^ (blue bars) and BK^-/-^ mice (red bars) applying an *in vitro* model of I/R at normoxia and reoxygenation (both at 21% O_2_) upon 90 min of anoxia**. (A)** Coupled respiration (OXPHOS) with complex I (CI)) substrates malate (2 mmol/l), glutamate (10 mmol/l) and complex II (CII) substrate succinate (10 mmol/l) in the presence of ADP (5 mmol/l) before and after 90 min of anoxia (OXPHOS90). **(B)** OXPHOS recovery ratio OXPHOS90/OXPHOS **(C)** Isolated activity of complex IV (cytochrome c oxidase (COX)) with redox substrates ascorbate (2 mmol/l) and TMPD (5 mmol/l) followed by sodium azide in the presence of complex III blocker Antimycin A (2 μmol/l). Data are mean±SEM for BK^+/+^ (n=7) and BK^-/-^ (n=6) with ns=non-significant difference between genotypes within the respective conditions.

**Figure S4: Hemodynamics of BK^+/+^ and BK^-/-^ hearts upon 5 min of ischemia and 5 min of reperfusion.**

**(A)** The schematic illustration of the protocol used for the assessment of coronary flow in isolated, perfused hearts of BK^+/+^ and BK^-/-^ mice. Upon 5 min of global zero-flow ischemia (green bar) coronary flow and perfusate volumes were determined within 5 min of reperfusion. **(B)** Time course of the reactive hyperemic response after equilibration (E) followed by 5 min of ischemia (I) and reperfusion (R). **(C)** Perfusate volumes were not different between both genotypes.

**Table S1: Summary of parameters assessed in BK^+/+^ and BK^-/-^ hearts subjected to the *ex-vivo* Langendorff preparation.**

Basal heart rate and coronary flow were measured over the last 2 minutes of the respective equilibration period (see Fig. 4A). Heart weights were determined at the end of the experiment. Data are mean±SEM for BK^+/+^ (n=6 or 5) and BK^-/-^ (n=7 or 5) groups with *P<0.05 indicating significant difference between genotypes within the respective conditions. Abbreviations used: (BW) body weight, (HW) heart weight.

**Figure S1**

**A**


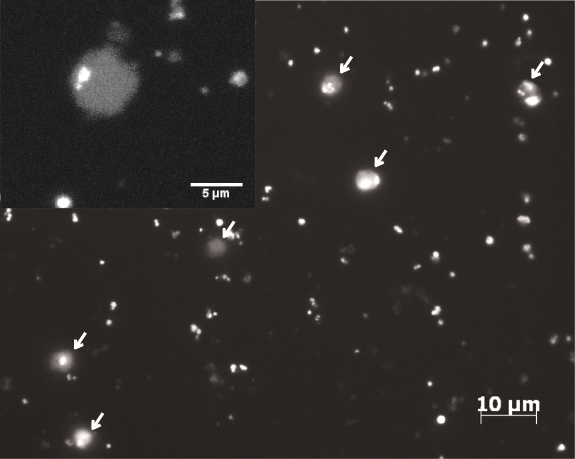

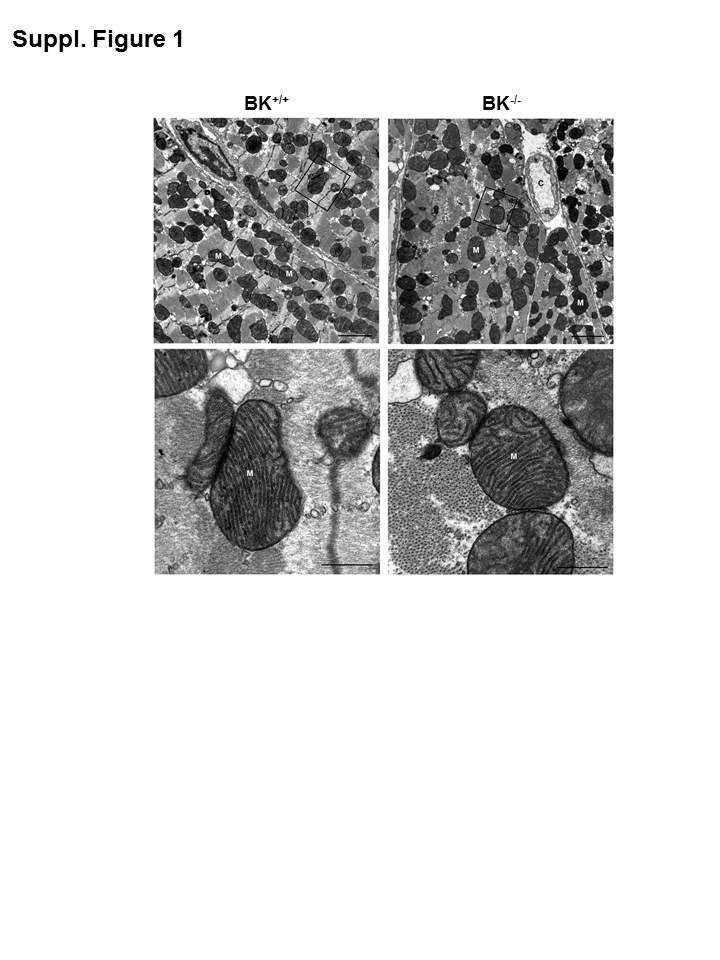


**B**

**Figure S2**


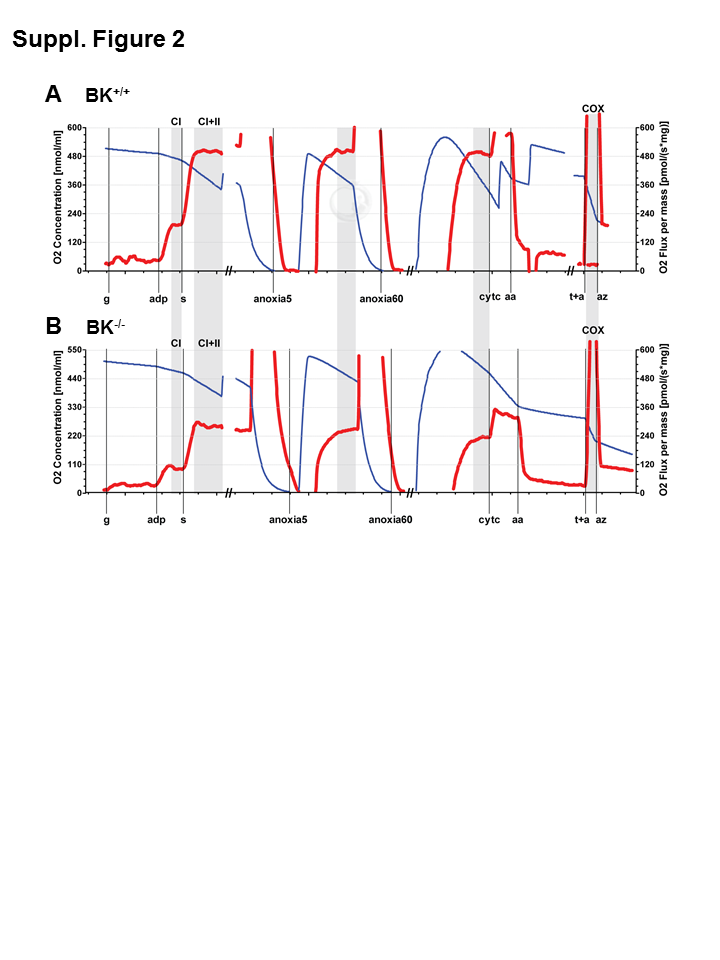


**Figure S3**

**
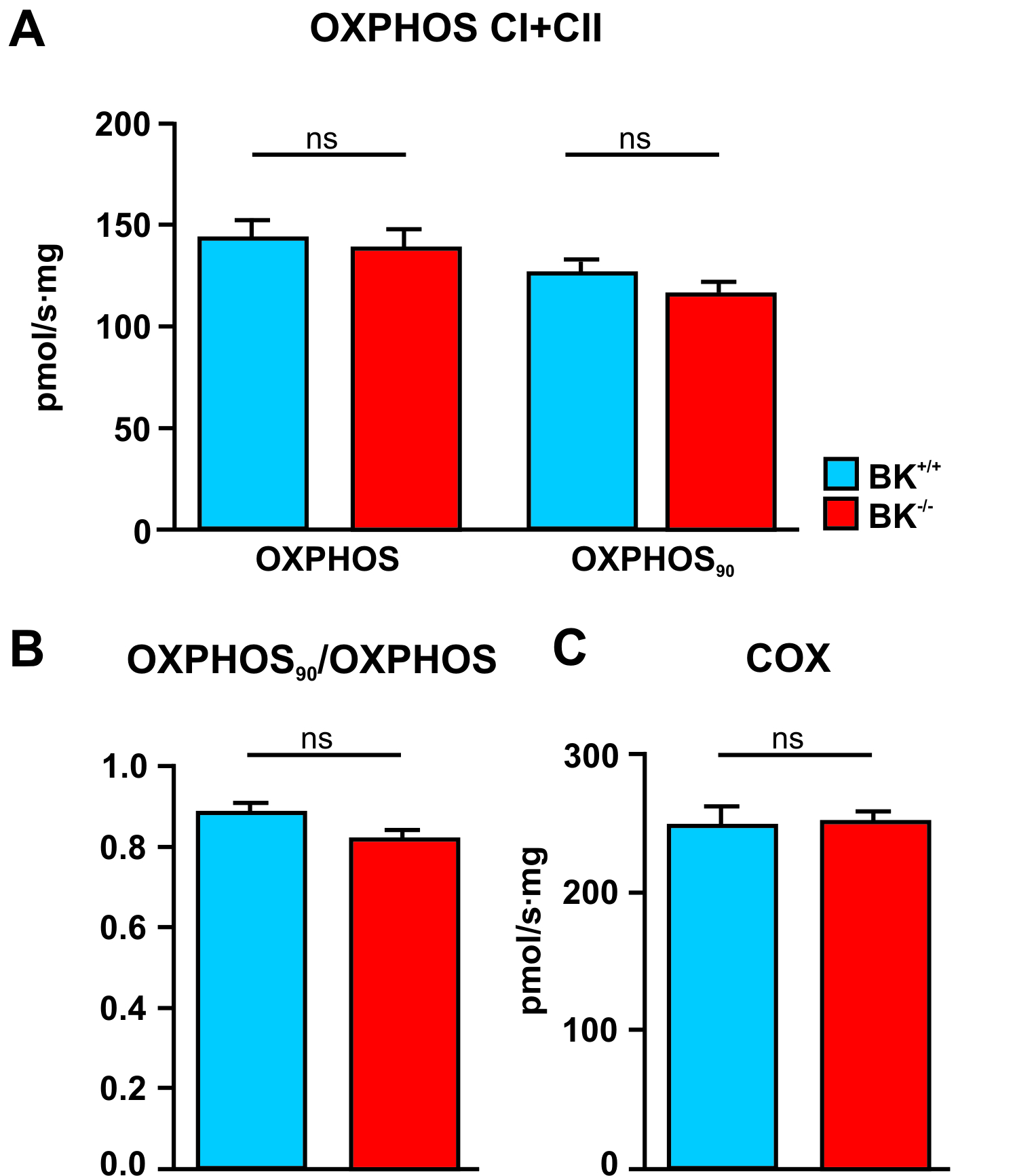
**

**Figure S4**

**
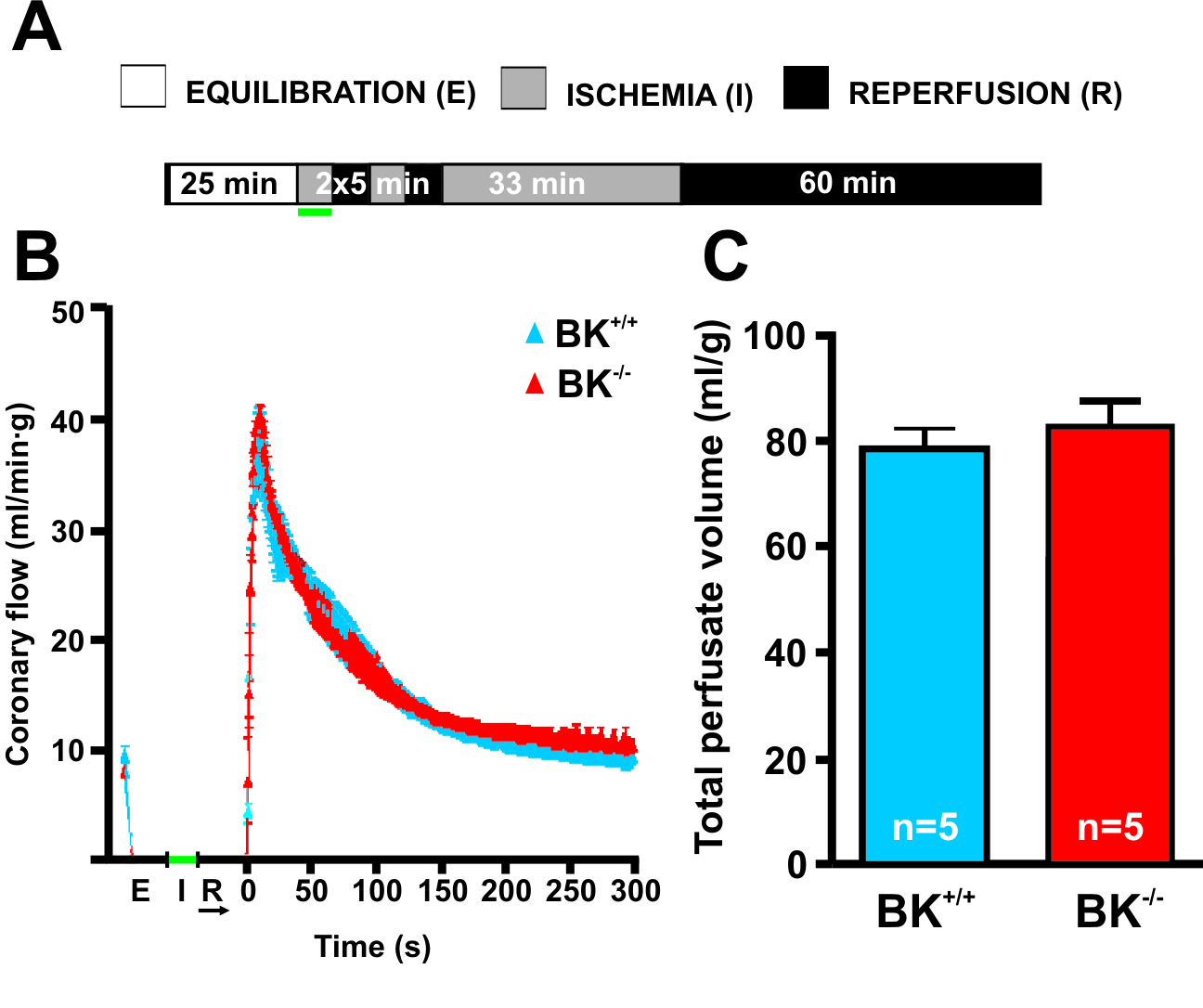
**

**Table S1**

|  | **CTRL BK^+/+^** | **CTRL BK^-/-^** | **BK^+/+^**  **(prior IP)** | **BK^-/-^**  **(prior IP)** |
| --- | --- | --- | --- | --- |
| **n** | 6 | 7 | 5 | 5 |
| **BW**  (g) | 32.31±3.00 | 25.66±1.12 | 33.48±2.72 | 27.74±1.67 |
| **HW**  (g) | 0.20±0.01 | 0.16±0.01***** | 0.20±0.01 | 0.16±0.01***** |
| **HW/BW∙1000** (g/kg) | 6.26±0.46 | 6.05±0.32 | 5.89±0.21 | 5.84±0.23 |
| **Time delay to cannulation**  (s) | 115±12 | 144±15 | 142±14 | 121±10 |
| **Coronary flow rate**  (ml/min) | 1.46±0.16 | 1.44±0.08 | 1.58±0.02 | 1.52±0.09 |
| **Coronary flow rate/HW** (ml/min∙g) | 7.37±0.68 | 9.21±0.49 | 8.05±0.72 | 9.39±0.74 |
| **Heart rate**  (bpm) | 358.5±22.7 | 360.4±16.7 | 334.6±23.2 | 332.7±18.0 |
